# T Cells Immunophenotyping and CD38 Overexpression as Hallmarks of the Severity of COVID-19 and Predictors of Patients’ Outcomes

**DOI:** 10.3390/jcm12020710

**Published:** 2023-01-16

**Authors:** Nesrin I. Tarbiah, Nuha A. Alkhattabi, Abdullah J. Alsahafi, Hani S. Aljahdali, Husam M. Joharjy, Maryam H. Al-Zahrani, Aliaa M. Sabban, Rana A. Alghamdi, Maha J. Balgoon, Reham A. Khalifa

**Affiliations:** 1 Biochemistry Department, Faculty of Science, King Abdulaziz University, Jeddah 21589, Saudi Arabia; 2 King Abdulaziz Hospital, Ministry of Health, Jeddah 23221, Saudi Arabia; 3 Department of Clinical Biochemistry, Faculty of Medicine, King Abdulaziz University, Jeddah 21589, Saudi Arabia; 4 Department of Chemistry, College of Sciences & Arts, King Abdulaziz University, Rabigh 21589, Saudi Arabia; 5 Medical Microbiology and Immunology, Faculty of Medicine, Ain Shams University, Cairo 11371, Egypt

**Keywords:** COVID-19, T cells, T-helper (CD4^+^), T-cytotoxic (CD8^+^), CD38

## Abstract

Background: By the end of 2019, the COVID-19 pandemic spread all around the world with a wide spectrum of clinical presentations ranging from mild to moderate to severe or critical cases. T cell subtype dysregulation is mostly involved in the immunopathogenic mechanism. The present study aimed to highlight the role of monitoring T cell subtypes and their activation (expression of CD38) in COVID-19 patients compared to healthy subjects and their role in predicting severity and patients’ outcomes. Materials: The study involved 70 adult COVID-19 confirmed cases stratified into three groups: a mild/asymptomatic group, a clinically moderate group, and a clinically severe/critical group. Flow cytometry analysis was used for the assessment of CD3^+^ cells for total T cell count, CD4^+^ cells for helper T cells (Th), CD8^+^ cells for cytotoxic T cells (Tc), CD4^+^CD25^+^ cells for regulatory T cells (T reg), and CD38 expression in CD4^+^ T cells and CD8^+^ T cells for T cell activation. Results: A statistically significant difference was found between COVID-19 cases and healthy controls as regards low counts of all the targeted T cell subtypes, with the lowest counts detected among patients of the severe/critical group. Furthermore, CD38 overexpression was observed in both CD4^+^ and CD8^+^ T cells. Conclusion: Decreased T cell count, specifically CD8^+^ T cell (Tc), with T cell overactivation which was indicated by CD38 overexpression on CD4^+^ and CD8^+^ T cells had a substantial prognostic role in predicting severity and mortality among COVID-19 patients. These findings can provide a preliminary tool for clinicians to identify high-risk patients requiring vigilant monitoring, customized supportive therapy, or ICU admission. Studies on larger patient groups are needed.

## 1. Introduction

By the end of 2019, a new strain of coronavirus, severe acute respiratory syndrome coronavirus 2 (SARS-CoV-2), was responsible for the COVID-19 pandemic outbreak, which has spread with an epidemiological scenario rapidly progressing into a worldwide crisis. Globally, as of September 2022, there were 613 million confirmed COVID-19 cases, resulting in 6.5 million deaths, reported to the World Health Organization (WHO) [1]. To properly manage the hospitalization of COVID-19 patients, it became obvious that the methods to assess the severity and outcome of the disease, such as the distribution of cell types and the viral infection in peripheral blood, were very important [2].

Most of the COVID-19 infections were mild. Unfortunately, serious complications occurred in about 25% of patients, including multiple organ failure, acute respiratory distress syndrome (ARDS), and even death [3,4]. Patients with COVID-19 can either be directly damaged by the pathogen itself or by the pathogen triggering an excessive immune response [5].

Several hematological and biochemical parameters were suggested as disease progression markers. Inadequate clinical outcomes have been associated with thrombocytopenia, lymphopenia, elevated neutrophil-to-lymphocyte ratio, neutrophilia, elevated creatine, and D-dimer, along with others [6,7,8]. Moreover, the life-threatening proinflammatory response induced by SARS-CoV-2 can eventually damage the lung endothelial cells, impairing gaseous exchange [4]. ARDS is caused by the immune response imbalance caused by the extreme release of inflammatory cytokines, also known as cytokine storm syndrome (CSS) [9].

The pivotal role of the immune system in COVID-19 pathophysiology involves helping the host’s viral defense in the early stages. Then, in the more severe stages, it can act as an important driver for disease condition deterioration [10]. The immune dysregulation associated with COVID-19 involves changes in both the absolute count of lymphocyte subsets and functional status differences, which is associated with unsuccessful elimination of the virus and extreme inflammation [11]. For critical patients, the excessive inflammatory responses combined with the overactivation of different lymphocyte subsets and the subsequent apoptosis, anergy, and cell exhaustion could help to explain the course of the disease [12].

T cells play a vital role in viral clearance [13,14]. However, persistent virus stimulation results in T cell exhaustion and reduced function [15,16]. It has been shown that COVID-19 patients had reduced CD4^+^ and CD8^+^ T cell counts in peripheral blood. The cells were found to be hyper-activated with high cytotoxic granule concentration, which could help mediate cytokine release syndrome (CRS) [17].

One feature that was regularly reported in COVID-19 patients was lymphocytopenia with higher selectivity for T cell lineages [18]. In addition to the imbalance between neutrophil and lymphocyte numbers, both populations of cells also showed increased fluorescence signals, which reflects their activation status and can be used as an independent predictor for the need for mechanical ventilation or death among COVID-19 patients [19]. Lymphopenia appears to be more common in severe COVID-19, which may reflect lymphocytes’ adhesion to inflamed respiratory vascular endothelium or recruitment to the respiratory tract [20].

In COVID-19, it has been suggested that the well-established balance between the expression of inhibitory and excitatory markers could impact the disease progression. Thus, prolonged T cell activation encountered during SARS-CoV-2 infection and reduced downregulation of the immune response can lead to the production of the cytokine storm [21].

Viral infection triggers different pathways that can promote inflammatory conditions, such as CD38 activation. Recently, Horenstein et al. (2021) highlighted that the possible role of CD38 in COVID-19 pathogenesis includes regulation of immune cell migration to the inflamed site, induction of different cytokines causing their secretion, and nucleotidase enzyme activity, which can augment lung immunopathology and result in a cytokine storm. CD38 has also been shown to be involved in cell adhesion and immune cells being uncontrollably activated, which could contribute to thrombosis and lymphopenia [9]. CD38 interacts with its counterreceptor, CD31 (endothelial/platelet cell adhesion molecule-1), which has two major consequences: thrombosis and lymphopenia, which are both COVID-19 disease severity predictors [22].

Most elderly patients develop severe COVID-19, which makes the high morbidity rate in the elderly a significant COVID-19 feature [23,24]. Aging is also characterized by increased immune cell CD38 expression [25]. This can worsen the cytokine storm, leading to fatal ARDS, which is commonly found in older COVID-19 patients [26].

Ultimately, CD38, as an orchestrating immune-modulatory enzyme, provides a potential target involved in COVID-19 pathogenesis. Different pharmacological approaches can be used to target CD38, including enzyme-modulating monoclonal antibodies and small-molecule inhibitors [27]. Thus, targeting CD38 enzymatic activities may contribute to designing novel therapeutics which would help alleviate the detrimental COVID-19 effects.

The aim of this study is to highlight the role of monitoring T cell subtypes and their activation (expression of CD38) in COVID-19 patients compared to healthy subjects and their role in predicting severity and patients’ outcomes.

## 2. Materials and Methods

### 2.1. Patients

A case-control study was performed on 70 adult patients diagnosed with COVID-19, confirmed cases on a clinical, radiological, and laboratory basis according to the diagnostic guidelines of the Saudi Ministry of Health. They were admitted to King Abdulaziz Hospital in Jeddah Saudi Arabia between March 2021 and July 2021. Fourteen [14] age- and sex-matched individuals who were apparently healthy were included in this study as a control group.

Inclusion criteria: The patients were admitted to King Abdulaziz hospital, where the blood samples were collected. The patients were clinically and radiologically diagnosed as COVID-19 cases and were confirmed in the Regional Laboratory, Jeddah, KSA through positive testing of respiratory samples for SARS-CoV-2 by real-time reverse transcriptase-polymerase chain reaction (RT-PCR) using LightCycler 480 II Roch, Germany. The control group included healthy healthcare workers who had a negative screening for SARS-CoV-2 using RT-PCR. The limited size of the control group was attributed to the limited number of healthy controls available during the pandemic (those not infected with COVID-19 and not vaccinated), as most of the population were currently or previously COVID-19-infected.

The patients were clinically classified according to the National Health Commission of China’s New Coronavirus Pneumonia Prevention and Control Program July 2020 [28], as follows: mild cases: mild clinical symptoms, with no signs of pneumonia on the imaging examination; moderate cases: showing respiratory symptoms and fever, accompanied by manifestations of pneumonia during imaging; severe cases: for adults, the cases met any of these criteria: oxygen saturation ≤ 93% at rest, shortness of breath with a respiratory rate of ≥ 30 times/min, or an arterial oxygen partial pressure (PaO2)/oxygen concentration (FiO2) ≤ 300 mmHg (1 mmHg = 0.133 kPa); critical cases: any cases that met any of the following criteria: shock, respiratory failure which requires mechanical ventilation, or another organ failure that requires admission to the intensive care unit (ICU). Patients were followed up for the determination of outcome as survivors (hospital discharge) or deceased (in-hospital death).

Patient groups were divided as follows:

Group 1: Controls: Included 14 age- and sex-matched apparently healthy individuals.
Group 2: Mild group: Included 22 asymptomatic and clinically mild laboratory-confirmed COVID-19 cases with positive SARS-CoV-2 RT-PCR testing.
Group 3: Moderate group: Included 22 moderate laboratory-confirmed COVID-19 cases.
Group 4: Severe group: Included 26 severe and critical laboratory-confirmed COVID-19 cases.

Exclusion criteria were chronic infections (HCV, HBV), cancers, any immunological disorders or patients on immunosuppressive drugs or chemotherapy, any underlying hematological disorder, and laboratory and clinical signs of other infections that were not COVID-19.

### 2.2. Ethical Considerations

The study was performed after approval of the Research and Studies Department—Jeddah Health Affairs Institutional Review Board (IRB), registration number with KACST, KSA: H-02-J-002 research number 1373 in March 2021 and in accordance with the code of ethics of the World Medical Association (Declaration of Helsinki) and Good Clinical Practice guidelines. Patients or guardians were informed, and their informed consent was obtained before specimen collection.

### 2.3. Laboratory Work and Data Collection

SARS-CoV-2 laboratory confirmation was defined as a positive result of RT-PCR assay from nasopharyngeal swabs. Patients could then be classified into severity classes based on ICU admission, oxygen requirements, and clinical data. Patient’s clinical data were extracted from the electronic medical record, and laboratory data were extracted from the date closest to that of research blood collection.

Laboratory data including total leukocytic count, neutrophil count, lymphocyte count, eosinophil count, neutrophil/lymphocyte ratio (NLR), platelet count, hemoglobin blood concentration, serum creatinine (S. Cr), aspartate transaminase (AST) serum level, alanine aminotransferase (ALT) serum level, and blood urea nitrogen (BUN) levels in the serum were collected from the patients’ electronic medical records within 7 days from confirmed nasopharyngeal swab results.

### 2.4. Flow Cytometry Analysis of T Cell Subtypes

The following cell surface molecules were detected: CD3^+^ cells for total T cell count, CD4^+^ cells for helper T cells (Th), CD8^+^ cells for Tc cells, CD4^+^CD25^+^ cells for regulatory T cells (T reg), and CD4^+^ T cell and CD8^+^ T cell CD38 expression for T cell activation assessment.

Blood samples were collected in a Na-Heparin tube (3 mL) from all study subjects and sent immediately for flow cytometric analysis. Each sample was diluted 1:1 in PBS. This was followed by adding the diluted blood sample on Histopaque to make up the total amount of 60% diluted sample. Next, buffy coats were collected after centrifugation and then washed twice with PBS.

Samples were prepared for flow cytometry analysis by adding the target fluorescent antibodies at the recommended dilution by the manufacturers, as follows: anti-CD3-FITC (20019174, DAKO), anti-CD4-FITC (20010865, DAKO), anti-CD8-APC (20024877, DAKO), anti-CD25-PE (341011, BD), and anti CD38 (345806, BD). This was followed by incubation at room temperature in the dark for 30 min. Finally, samples were analysed using flow cytometry FACS Aria 3 from BD company followed by data analysis using FACSDiva version 9 software (BD Biosciences, San Jose, CA, USA).

Lymphocytes were gated according to light scatter parameters that reflect cell morphological characteristics (forward scatter reflecting cell size, side scatter reflecting internal structure of cell). At least 10,000 events were assessed for each sample. The percentage of target cells, absolute count of every target cell per 10,000 events, and median of fluorescence intensity (MFI) values were used for further analysis.

**Figure 1:**
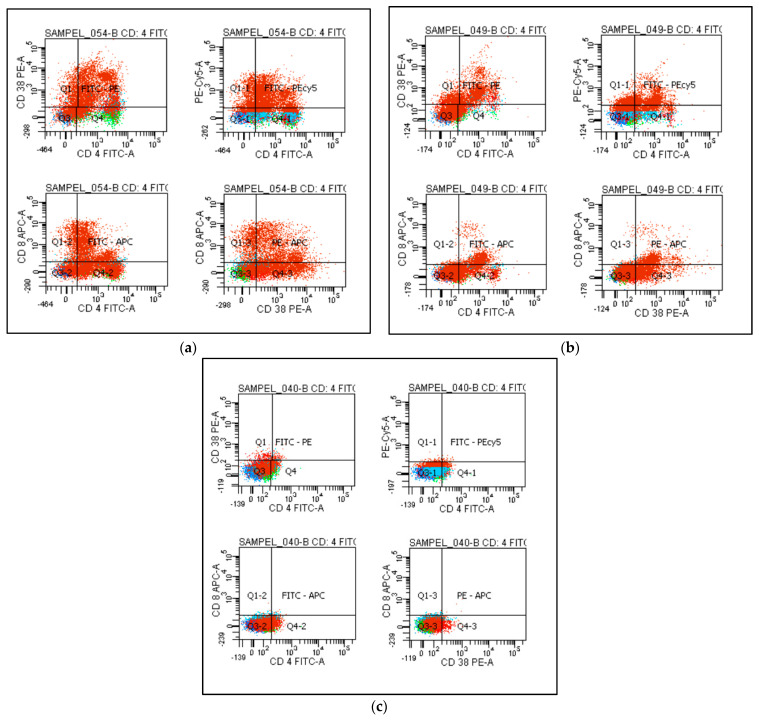
Co-expression of CD38 on CD4^+^ T cells and CD8^+^ T cells. An example of flow cytometry analysis showing the expression of CD38 on both CD4 and CD8 T cells is represented in different groups up of patients as (**a**) mild group, (**b**) moderate group, and (**c**) severe group.

CD38 expression was measured on their surface (nonparametric histogram) as an MFI. Representative flow cytometry plots (Figure 1) show mild, moderate, and severe groups.

### 2.5. Statistical Analysis

The collected data were revised, coded, tabulated, and introduced to Prism GraphPad software and Statistical Package for Social Science (SPSS 25). Results are presented as mean ± SE describing both cell percentages and total count. A normality test was performed to check the normal distribution, then an ANOVA test was used to compare differences between all groups followed by a *t*-test for the comparison of two groups for normal distribution parameters, while Kruskal–Wallis and Mann–Whitney tests were used for parameters that were not normally distributed. A *p*-value < 0.05 was considered significant, ** *p*-value < 0.01 was considered very significant, *** *p*-value < 0.001 and **** *p*-value < 0.0001 were considered extremely significant.

Furthermore, the chi-square test was used to examine the relationship between two qualitative variables. Fisher’s exact test was used to examine the relationship between two qualitative variables when the expected count was less than 5 in more than 20% of cells. A post hoc test was used for comparisons of all possible pairs. A scatter diagram was used to show the correlation between CD38 expression in Th CD4^+^ T cell and Tc CD8^+^ T cell subtypes. Receiver Operating Characteristics (ROC) analysis was used to evaluate the predictive value of CD38 expression for mortality among the severe/critical patient group.

## 3. Results

Table 1, Table 2 and Table 3 show the demographic data of the participants as age, sex, and other data collected, including laboratory data within 7 days from confirmed nasopharyngeal swab results. The mean age of patients was 51.04 ± 15.68 (40 males and 30 females), and the mean age of controls was 46.27 ± 7.20 (seven males and seven females) (Table 1). No statistically significant difference was observed between cases and controls regarding age and sex distribution (Table 2). Participants were classified into four groups: Group 1 *(n = 14)* included healthy controls, group 2 included 22 mild *(n = 18)* and asymptomatic *(n = 4)* cases, group 3 included 22 clinically moderate cases, and group 4 included 26 clinically severe *(n = 9)* and critical *(n = 17)* cases. Among patients of group 4, 17 patients required ICU admission (eleven patients were mechanically ventilated, six patients required continuous positive airway pressure (CPAP) ventilation, and eleven patients died (non-survivors)). One patient expired among patients of group 3, while no expired patients were detected among patients of group 2. A statistically significant difference was found between cases and controls regarding WBCs, neutrophils, lymphocyte count, neutrophil-to-lymphocyte ratio (NLR), AST, ALT, S. Cr., and BUN serum levels. No statistically significant difference was detected as regards eosinophils, platelet count, and hemoglobin blood level (Table 2). A statistically significant difference was found between cases and controls as regards patients with eosinopenia, where 41 out of 70 patients (58.57%) showed decreased eosinophil count (Table 3).

**Figure 2:**
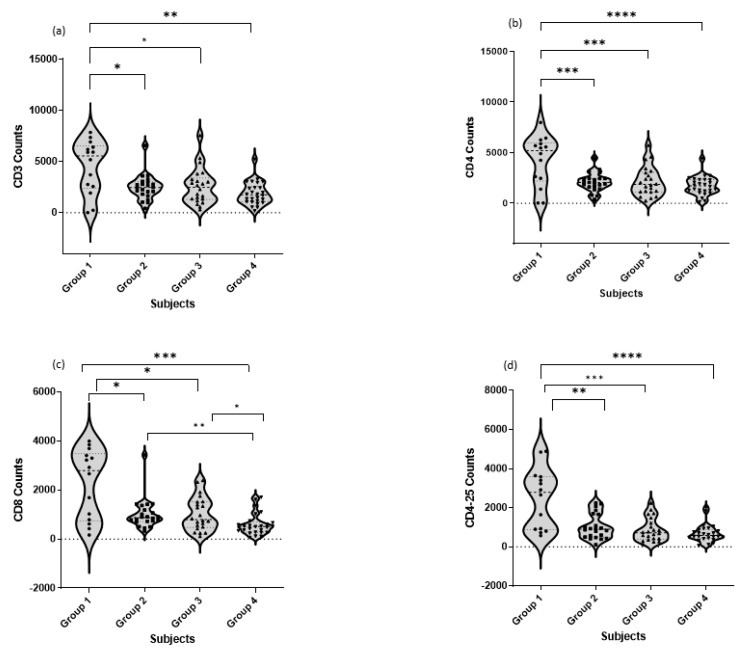
Comparison of cell counts of total CD3^+^ T cells (Mann–Whitney) and different sub types: Th CD4^+^ (*t*-test), Tc CD8^+^ cells (Mann–Whitney), and Treg CD4^+^CD25^+^ cells (Mann–Whitney) in different COVID-19 patients. Data represent the cell count of (**a**) CD3^+^, (**b**) CD4^+^, (**c**) CD8^+^, and (**d**) CD4^+^CD25^+^ T cells in mild/asymptomatic, moderate, and severe/critical COVID-19 patients. For all analyzed T cell subtypes, a significant difference was recorded between healthy control and COVID-19 groups of patients. * *p*-value < 0.05 is considered significant, ** *p*-value < 0.01 is considered very significant, *** *p*-value < 0.001 and **** *p*-value < 0.0001 are considered extremely significant.

According to the collected data, both percentages (Table 4) and absolute counts (Figure 2) of different studied T cell subtypes including CD3^+^ cells, CD4^+^ cells, CD8^+^ cells, CD4^+^CD25^+^ cells, CD38^+^ cells, CD4^+^CD38^+^ cells, and CD8^+^CD38^+^ cells in different patient groups showed a significant decrease between healthy control and COVID-19 patient groups.

Figure 2 represents the comparison of cell counts of total CD3^+^ T cells and different subtypes, Th CD4^+^, Tc CD8^+^, and Treg CD4^+^CD25^+^, in different COVID-19 patients. There was a significant difference between the asymptomatic and severe groups as well as moderate and severe groups in the Tc CD8^+^, where it decreased in the severe group when compared to the other COVID-19 groups. Moreover, T reg CD4^+^CD25^+^ subtype was significantly decreased in the patient group compared to the healthy group.

**Figure 3:**
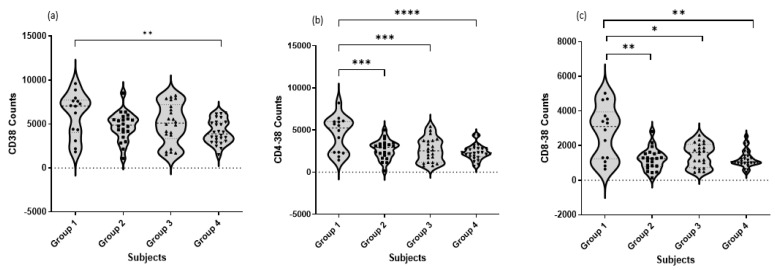
Comparison of cell counts of total CD38^+^ (*t*-test) and different T cell subtypes, Th CD4^+^ (*t*-test) and Tc CD8^+^ cells (Mann–Whitney), in different COVID-19 patients. Data represent cell counts of (**a**) CD38^+^, (**b**) CD4^+^CD38^+^, and (**c**) CD8^+^CD38^+^ T cells in mild/asymptomatic, moderate, and severe/critical COVID-19 patients. For all analyzed T cell subtypes, a significant difference was recorded between healthy control and COVID-19 groups of patients. * *p*-value < 0.05 is considered significant, ** *p*-value < 0.01 is considered very significant, *** *p*-value < 0.001 and **** *p*-value < 0.0001 are considered extremely significant.

Our study showed that CD38 expression was also detected in different T cell subtypes, CD8^+^ T cells and CD4^+^ T cells, as represented in Figure 3. In general, comparing the healthy control group with COVID-19 patients, the results showed that there was a significant difference in the count of cells expressing CD38^+^, and severe patients’ count was the lowest.

The expression of CD38^+^ was detected as CD4^+^CD38^+^ subtype and showed a highly significant difference. Different COVID-19 patients in asymptomatic/mild, moderate, and severe/critical groups also showed significant differences, as a decrease in the count was recorded when comparing them with the healthy control group, with *p* < 0.001, *p* < 0.001, and *p* < 0.0001, respectively, and the lowest count of the CD4^+^CD38^+^ subtype was recorded in the severe group.

Finally, the expression of CD38 on Tc was also recorded as the CD8^+^CD38^+^ subtype and showed a highly significant difference. Different COVID-19 patients in asymptomatic/mild, moderate, and severe/critical groups also showed significant differences, as a decrease was recorded comparing them with the healthy control group, with *p* < 0.01, *p* < 0.05, and *p* < 0.01, respectively, and the lowest count of the CD8^+^CD38^+^ subtype was also recorded in the severe group.

**Figure 4:**
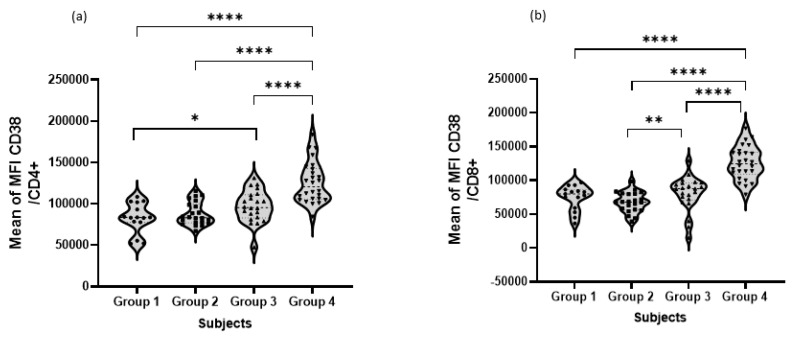
Comparison of CD38 expression (mean MFI) in (**a**) Th CD4^+^ T cell (*t*-test) and (**b**) Tc CD8^+^ T cell (Mann–Whitney) subtypes between controls and different COVID-19 patients. Data represent a statistically significant increase in CD38 expression among the patients of the severe group in both T cell subtypes. * *p*-value < 0.05 is considered significant; ** *p*-value < 0.01, **** *p*-value < 0.0001.

Table 5 shows a statistically significant difference between the moderate and the severe groups of patients compared to the mild group of patients as regards age, lymphocyte count, and ALT serum level. The older age group was among the patients of the moderate and severe groups. A statistically significant increase is shown among patients of the severe group compared to the mild and the moderate groups as regards WBC count, neutrophil count, NLR, S. Cr., BUN serum level, and mean MFI CD38 expression in both CD4^+^ and CD8^+^ T cells (Figure 4). Furthermore, a statistically significant decrease is shown among patients of the moderate group compared to the mild group as regards eosinophil count.

Among the three studied groups, a statistically significant difference is shown as regards gender distribution, where 13 patients (59.09%) and 22 patients (84.62%) were males among moderate and severe case groups, respectively, (Table 6). Table 6 also shows a statistically significant difference between the patients of the severe group as regards the number of patients with increased WBC count (61.5%), increased BUN serum level (84.62%), and the number of deceased cases (42.31%) compared to the patients of both the moderate and the mild groups. A statistically significant difference is shown between the patients of both the moderate and the severe groups as regards the number of patients with decreased eosinophil count (31.82% and 57.69%, respectively) and increased AST (27.27% and 57.69%, respectively) and ALT (31.82% and -%, respectively) serum levels compared to the patients of the mild group. As regards the number of patients with decreased lymphocyte count, a statistically significant difference is found between the patients of the severe, moderate, and mild groups (84.62%, 31.82%, and 0%, respectively).

**Figure 5:**
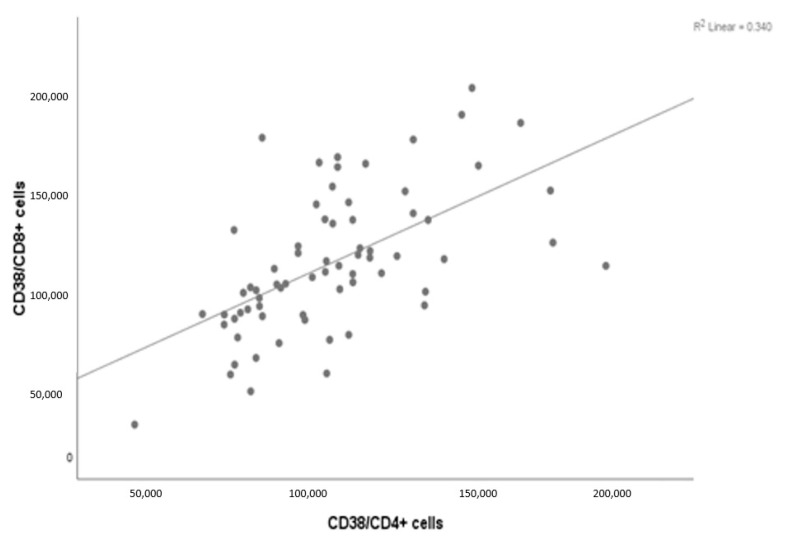
Scatter diagram showing the correlation between CD38 expression in Th CD4^+^ T cell and Tc CD8^+^ T cell subtypes.

With regard to the scatter diagram, Figure 5 showed a significant correlation between CD38 expression in Th CD4^+^ T cells and Tc CD8^+^ T cells (r = 0.583, *p* < 0.001) (Table 7).

**Figure 6:**
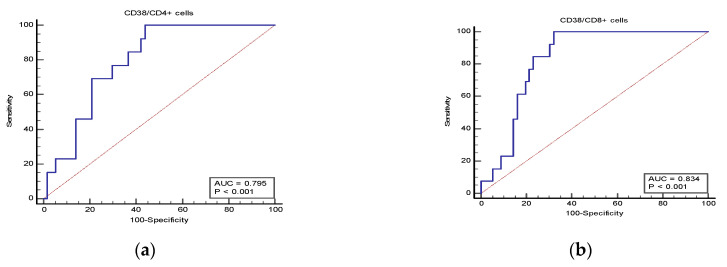
ROC curve: (**a**) CD38/CD4^+^ cells; (**b**) CD38/CD8^+^ cells to predict mortality among patients of the three studied groups.

To predict the mortality among patients, Receiver Operating Characteristics (ROC) analysis was performed on patients of the three studied groups (Figure 6a,b and Table 8), which demonstrates the value of CD38 expression (MFI) in both T cell subtypes as a predictor of mortality among the three studied groups, where CD38 expression in CD4^+^ T cells had an area under the curve (AUC) of 0.80, standard error (SE) of 0.06, and confidence interval (CI) of 0.687–0.887; for CD8^+^ T cells, the AUC was 0.83, the SE was 0.05, and the CI was 0.725–0.913 for predicting mortality. Table 9 shows that, based on the Youden index calculation, the cut-off values for CD38 expression in CD4^+^ T cell and CD8^+^ T cell subtypes that showed the highest sensitivity to predict mortality and the highest negative predictive value were >100.87 × 10^3^ and >91.46 × 10^3^, respectively.

**Figure 7:**
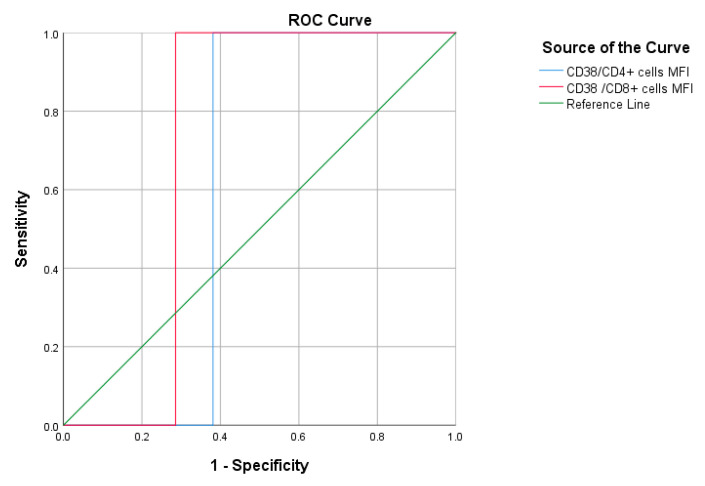
ROC curve of CD38/CD4^+^ cells and CD38/CD8^+^ cells to predict mortality among patients of the moderate group.

**Figure 8:**
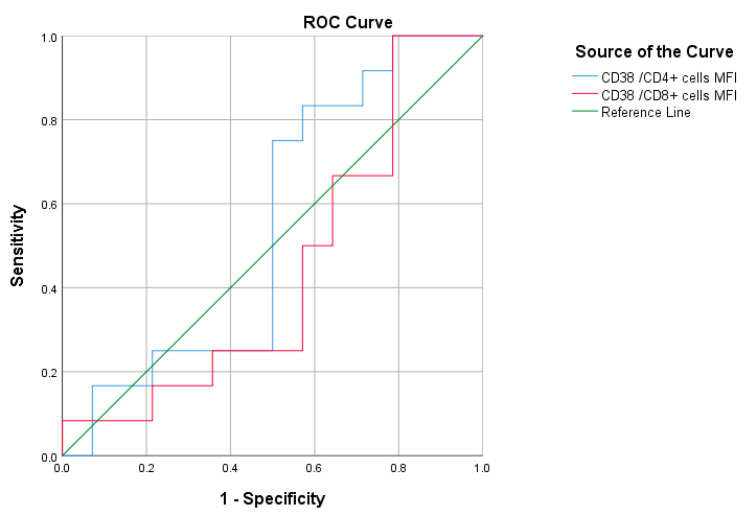
ROC curve of CD38/CD4^+^ cells and CD38/CD8^+^ cells to predict mortality among patients of the severe/critical group.

ROC analysis was performed on patients of the moderate group **(**Figure 7 and Table 10) and patients of the severe/critical group (Figure 8 and Table 11) separately; the results showed no statistically significant predictive value.

## 4. Discussion

Immune dysregulation has been involved in the pathogenesis of SARS-CoV-2 infection. Patients with COVID-19 can experience the disease in many forms, ranging from mild or even asymptomatic to severe, which requires hospitalization with mechanical ventilation that can result in a high fatality rate [29]. Some severe COVID-19 patients present with ARDS, which results in severe respiratory damage. During viral infections with acute respiratory effects, the observed pathology can be a result of the virus causing direct effects, the indirect result of the triggering of the immune response to be overaggressive, or both [30]. For severe COVID-19 patients, the role characteristics of the immune response and how the responses can be related to clinical aspects of the disease have not been sufficiently determined. In the presented study, T cell subtypes and the expression of CD38 was assessed and correlated to the severity of COVID-19 and patients’ outcomes.

It is suggested that respiratory viral infections may cause pathology through an immune response that is too strong, causing immunopathology [31], or as explained in other studies, through a mechanism involving T cell exhaustion or dysfunction [32,33].

Even though it has been suggested that in patients with COVID-19 there is T cell activation [34], some studies have shown a decrease in T cell functions or cytotoxicity [35], and other studies have not seen these changes [32]. Currently, it remains unclear how T cell activation during COVID-19 lymphopenia should be viewed [36].

CD38 is an ectoenzyme with versatile immunological functions, which is recognized commonly as a hallmark of activation of immune cells. It is also considered a nucleotidase linking NAD^+^ metabolism and the immune system via adenosinergic signaling, Ca^2+^ second messengers, leukocyte migration, and epigenetic regulation [9]. When SARS-CoV-2 reaches the site where it causes infection, CD38 may be involved in direct Ca^2+^ signaling, which has been shown to be important for viral endocytosis, regulating interferon-stimulated genes (ISGs), enhancing antiviral oxidative bursts from macrophages, orchestrating the deadly cytokine storm or hyperinflammatory response, and modulating exoenzymatic adenosinergic networks. During the immunopathogenesis of COVID-19, this may cause the accumulation of immune cells in the lungs and may culminate in a likely CD38-mediated thrombosis [37].

The present study was conducted from March to July 2021. Phylogenic data of SARS-CoV-2 variants prevalent among the study group were not available. However, the study of Obeid et al. (2021) reported that SAR-CoV-2 virus was introduced to Saudi Arabia in February 2020 with the D614G spike mutation present. However, between February and August 2020, increasing numbers of patients infected with the wild-type virus were also reported. The most common variants detected were the NSP12_P323L mutation 94.9%, followed by the D614G mutation (76%) and the NS3_Q57H mutation (71.4%). D614G was associated with higher morbidities than the wild-type virus, including higher rates of death and hospitalization [38].

Another study, by the end of 2021, reported the prevalence of the Delta variant (40.9%), Beta variant (15.9%), and Alpha variant (11.6%) among 320 SARS-CoV-2 sequenced strains in Saudi Arabia [39]. The Delta variant is highly contagious, and it was suggested that the Delta variant might cause more severe illness than other strains in unvaccinated persons. It is characterized by the spike protein mutations T19R, ∆157–158, L452R, T478K, D614G, P681R, and D950N; several of these mutations may affect immune responses directed toward the key antigenic regions of the receptor-binding protein [40].

Seventy laboratory-confirmed COVID-19 cases (40 males and 30 females) with a mean age of 51.04 ± 15.68 were included in the present study. Among the severe group of patients, 65.3% required ICU admission, 42.3% were mechanically ventilated, 23% required CPAP ventilation, and 42.3% died. A high case fatality rate among patients of the severe group was attributed to multiple organ system failure, as was evident by elevated liver function tests and renal function tests (statistically significant elevation was observed), or progressive respiratory distress, as was evident by the need for mechanical ventilation (42.3%). Only one patient died among patients of the moderate group, with a case fatality of 4.5% and a 100% survival rate among patients of the mild and asymptomatic group.

In the presented data, immunophenotypic investigation showed that there was a significant decrease in all studied T cell subtype counts and percentages with increased severity of COVID-19. Looking into the different COVID-19 groups in the study, severe patients showed the lowest obtained count of all the studied T cell subtypes, which was also associated with the highest fatality rate. The lowest counts were detected among total CD8^+^ Tc cells, CD4^+^CD25^+^ Treg cells, and activated Tc CD8^+^ T cells (CD38^+^CD8^+^).

In agreement with these data, the study by Wang and colleagues (2020) showed that the total number of CD8^+^ T cells, CD4^+^ T cells, NK cells, and lymphocytes was found to decrease significantly in COVID-19 patients, with severe cases having the lowest levels, particularly for CD8^+^ T cells. This was explained by either viral attachment or immune injury caused by high inflammation or even as a result of lymphocyte exudation to patients’ lungs, explaining recorded lymphopenia [41].

In another study by Ashrafi et al. (2021) that was performed on 40 severe COVID-19 patients, their findings were in line with our findings, whereas during the study, nine patients (22.5%) died and sixteen patients (40%) were admitted to ICU. The patients who were deceased had lower T cell and CD4^+^ T cell count when compared with patients who survived at the time of admission [42]. A marginally significant correlation was also shown between mortality and CD4 levels below 200/μL, but there were no significant associations found for the other variables observed and admission to the ICU. Additionally, in patients with aberrant CD38 expression (higher than 30%) or CD7 loss on T cells, there was a higher risk of mortality. The cases that ended in death rapidly progressed to refractory metabolic acidosis, ARDS, coagulopathy, septic shock, and finally multiple organ failure. These findings of lower lymphocyte counts were recorded in other studies as well [43,44,45,46].

Zheng et al. (2020) evaluated the clinical parameters of 67 noncritical and 32 critical COVID-19 cases and reported that patients who were critically ill had significantly lower counts of CD8 and CD4. However, in contrast with the findings of the present study, they observed lower neutrophil count in critically ill patients. Based on these results, it has been suggested that markers such as these could be useful for evaluating patient prognosis [47].

In a study by Chan and colleagues (2020), they clarified that generally, patients with COVID-19 had significantly lower levels of total lymphocytes, however to the contrary of the present observation, CD4^+^cells count and CD4/8ratio in their study were not significantly different among patients according to severity [48]. Similarly, Bobcakova et al. (2022) observed that there was a significantly higher CD8^+^ CD38^+^ cell count in non-survivors when compared to those who survived, both at admission and after hospitalization for one week; this may be caused by an initial higher viral load in non-survivors. It is suggested that the decline in innate immunity in older patients and their ability to control viral infection in the early stages could be the result of excessive inflammation, overactivation of lymphocytes, exhaustion, and apoptosis, explaining disease progress in critical patients [49].

When CD8^+^ and CD4^+^ T cell activation was assessed by CD38 expression in both cell types, the present results showed a statistically significant increase in expression of CD38 in both cell types among all patient groups compared to healthy controls, reflecting a state of overactivation. This agreed with the work by Sekine and others (2020), who investigated the SARS-CoV-2 humoral and cellular immune responses in acute, moderate, or severe patients with COVID-19 [43]. They reported that activation of T cells, demonstrated by the CD38 expression, was evident for patients with acute COVID-19, which is comparable to the results of previous studies [4,34,50].

Similarly, Mathew and colleagues (2020) observed that among patients with COVID-19, activation levels of CD8 and CD4 T cells (via HLA-DR and CD38 co-expression) were similar to the antiviral responses that have been observed for other infections [21]. Nevertheless, around 20% of the patients only had low levels of T cell activation when compared to the controls. The disease-severity-associated immunotype resulted in a robust activation of CD4^+^ T cells, a circulating follicular Th cell reduction, and exhausted or hyperactivated CD8^+^ T cells. Moreover, the immunotype that exhibited less CD4^+^ T cell activation was not directly associated with the severity of the disease, which suggested that during COVID-19, a less vigorous immune response could be associated with a pathology that was less severe. This was also observed in a subset of patients who had a high polymorphonuclear leukocyte count, which has been previously described [11,51]. Thus, it was concluded that most acute viral infections induce activation and proliferation of CD8^+^ T cells, which were detectable by HLA-DR and CD38 co-expression [52,53].

Clavarino et al. (2022) showed that low levels of activation of T cells can be associated with an improved disease outcome [54]. Exhibiting a high level of CD8^+^ and CD4^+^ T cell activation, marked CD8^+^ T cell lymphopenia, and increased levels of CD8^+^ T cell senescence was associated with a higher mortality rate. They observed a mortality of 26.1% among a cluster of patients with very high CD8^+^ and CD4^+^ activation in which 47.8% were patients with severe COVID-19. This was also confirmed in another study where they concluded that in infection with SARS-CoV-2, lymphocytopenia is a significant feature and that CD8^+^ and CD4^+^ T cells are overactivated, as demonstrated by the expression of HLA-DR/CD38 resulting in dysregulation of NAD^+^ metabolism [55]. However, in contrast with the present data, Tang et al. and Miller et al. (2020) reported that CD38 expression was significantly reduced in severe COVID-19 patients and those with higher mortalities [56,57]. Conflicting results could be due to group differences in the studied populations, the stage of the disease investigated, therapeutic lines, and even the variable definitions of the severity of the disease adopted by clinicians.

Data from the present study also showed a difference that was statistically significant between the moderate and the severe groups of patients compared to a mild group of patients as regards age, lymphocyte count, and ALT serum level. The older age group was observed among the patients of the moderate and severe groups. For the three study groups, there was a statistically significant difference shown for gender distribution, where 13 patients (59.09%) and 22 patients (84.62%) were males among moderate and severe case groups, respectively.

The worldwide epidemiological distribution of SARS-CoV-2 infection showed a higher virus susceptibility among elderly individuals and those with age-related morbidities. The study of Julianna et al. (2022) clarified that those individuals were more likely to have a hyperimmune response characterized by multiple organ failure and refractory acute lung pathology [37]. CD38 enzymatic activity was shown to be involved in aged tissues in the process of “inflammaging”. Thus, the dysregulated immune response reported in older patients could be linked to unsuccessful virus clearance at infection onset, followed by excessive inflammation [11,15,58,59].

The relationship between the activation of CD38 and depletion of NAD^+^ was highlighted as an aging-related feature with an evident role as a COVID-19 modulator in the elderly [9]. It was proposed that infection with SARS-CoV-2 stimulates overexpression of CD38. CD38-generated metabolites, including ADPR, nicotinamide, cADPR, and NAADP, stimulate several pathways that finally aggravate a hyperinflammatory profile typical in patients with COVID-19. Thus, overexpression of CD38 and depletion of NAD^+^ could both be considered common features of aging with a consequent overload of Ca^2+^, diminished mitochondrial function, and chronic inflammation predisposing elderly individuals to severe infections with COVID-19 [37].

Similar gender distribution, compared to the present study, was observed by Ashrafi et al. (2021), as regards the patients admitted to ICU [42]. They noted that for ICU admissions, there were significantly higher numbers of males than females. In the same context, the study by Conti et al. (2020) showed that females are less vulnerable to infection with COVID-19 due to immune receptors and immune system differences [60]. These sexual differences can be implicated in COVID-19 transmission, antiviral immune response, pathogenesis, and morbidity.

Estrogen has been proposed to regulate proinflammatory cytokine production and receptor response [60]. This might be attributed to the X chromosome in females having coding for immune-regulatory genes. Lower viral load was reported in women when compared to men. Furthermore, Sharma et al. concluded that differences based on sex could influence the outcomes for the patients regarding severity of the infection, viral load, and other comorbidities [61].

However, unlike the results of the present work, on comparing the data between patients as regards mortality and ICU admission, Ashrafi et al. (2021) found no significant differences between groups based on vital signs, age, and the time of starting symptoms [42]. Conflicting results could be caused by the diversity of different studied populations.

Furthermore, the present study demonstrated a significant correlation between expression of CD38 in Th CD4^+^ and Tc CD8^+^ T cells. These results were similar to the findings of Mathew et al. (2020), who stated that activation of CD4^+^ T cells was correlated with activation of CD8^+^ T cells [21]. Additionally, they found a correlation between CD38^+^ HLA-DR^+^CD4^+^ T cells and ferritin, renal insufficiency, acute kidney disease, and APACHE III score, indicating a relationship between disease severity and activation of CD4^+^ T cells.

A novel observation, for the present study, was demonstrated by the ROC Curve analysis showing the predictive value of expression of CD38 in both T cell subtypes among patients of the three studied groups for mortality, where expression of CD38 in CD4^+^ and CD8^+^ T cells had an AUC of 0.80 and 0.83, respectively, for predicting mortality. MFI cut-off values of >100.87 × 10^3^ and >91.46 × 10^3^ were estimated for the expression of CD38 in CD4^+^ T cells and CD8^+^ T cells, respectively, which showed 100% sensitivity and the highest negative predictive value for the prediction of mortality.

Various lymphocyte parameters could be used by clinicians to categorize patients during admission, particularly identifying patients with mild COVID-19 (T cell activation at low levels) or severe COVID-19 (with extreme CD8^+^T cell lymphopenia, a high level of CD8^+^ T cell senescence, and a high level of activation of CD8^+^ and CD4^+^ T cells). This is crucial for providing early and more appropriate treatment for different categories of COVID-19 patients [54].

Therapeutics targeting the CD38/NAD^+^ axis were highlighted as key options to improve COVID-19 patients’ outcomes. These included CD38 monoclonal antibodies and inhibitors which would modulate levels of NAD^+^ or vitamin B3 precursor administration (e.g., nicotinamide riboside, nicotinamide mononucleotide, nicotinamide), which would restore levels of NAD^+^ and the usual viral infection immune response [9].

Another concern for severe SARS-CoV-2 patients is secondary bacterial infections. CD38 was found to be essential during cytoskeleton rearrangements in phagocytes, the NAD^+^-dependent bacterial engulfment, and ADPR-dependent signaling needed for immune cells’ migration to the infection site [62,63]. Therefore, CD38 is considered to play a major part in potentiating an infection of SARS-CoV-2, as well as reacting to any secondary bacterial infections.

Thus, using prognostic factors to categorize patients infected with COVID-19 would be valuable to help identify those patients who could require admission to the ICU and provide appropriate effective treatments and supportive care for those patients [42]. Furthermore, this implies that more consideration should be given to patients with low CD4^+^ T cell counts who are in a critical condition, as they have an increased opportunistic infection risk, with lower levels of antiviral immune surveillance. The previous evidence appraises the clinical decision of providing targeted immunomodulatory therapies for patients with COVID-19 in the early stages of the disease, which can better support patients’ outcomes.

## 5. Conclusions and Recommendations

Ultimately, it was demonstrated that low T cell count, specifically Tc, as well as overactivation of T cells indicated by CD38 overexpression on CD4^+^/CD8^+^ T cells, had a prognostic role to predict mortality and severity among patients with COVID-19 and that these factors can shed light on the expected patients’ outcomes. However, due to the limited size of the studied group, these findings can provide only a preliminary tool for clinicians to identify high-risk patients requiring vigilant monitoring, customized supportive therapy, or ICU admission. Thus, studies on larger-scale populations and further characterization of T cell subtypes, including evaluation of CD38/HLA-DR co-expression, are necessary for more elucidation of these findings.

## Figures and Tables

**Table 1:** Study group distribution and participants’ demographic data.

Participants			N	%
Groups/ Age (First Quartiles, Median, Third Quartiles)	Group 1	Controls	14	17.6%
		Age (40.25) (44.5) (52)		
	Group 2	Mild or asymptomatic cases	22	25.9%
		Age (33.75) (38.5) (44.25)		
	Group 3	Moderate cases	22	25.9%
		Age (42.75) (54) (65.25)		
	Group 4	Severe or critical cases	26	30.6%
		Age (46) (63) (67.25)		
Gender	Male		47	55.9%
	Female		37	44.1%

**Table 2:** Comparison of the laboratory data measured between cases and controls.

	Controls	Cases	
	Mean ± SD	Mean ± SD	Reference Range
WBCs K/UL	6.01 ± 1.4	9.3 ± 4.99	4.5–10.5
NU K/UL	3.48 ± 1.32	7.45 ± 5.04	2.5–8
LY K/UL	2.17 ± 0.51	1.49 ± 1.03	0.9–5.1
NU/LY	1.74 ± 0.97	9.81 ± 13.84	-
EO K/UL	0.12 ± 0.13	0.14 ± 0.28	0.1–0.7
Plat. K/UL	288.73 ± 66.83	296.73 ± 104.84	150–450
HB gm/dL	12.19 ± 1.35	12.61 ± 1.89	12–15.5
AST U/L	23 ± 6.72	49.2 ± 39.47	0–50
ALT U/L	24.93 ± 10.05	47.89 ± 45.05	0–50
S. Cr Umol/L	58.87 ± 13.33	82.48 ± 42.74	53–123
BUN mmol/L	3.4 ± 1.05	6.84 ± 5.21	2.5–6.4

WBCs (leukocytic count), NU (neutrophil count), LY (lymphocyte count), EO (eosinophil count), NU/LY (neutrophil/lymphocyte ratio), Plat. (platelet count), HB (hemoglobin blood concentration), S. Cr (serum creatinine), AST (aspartate transaminase), ALT (alanine aminotransferase), BUN (blood urea nitrogen).

**Table 3:** Comparison of the gender and the laboratory data distribution between cases and controls.

		Controls	Cases
		N (%)	N (%)
Gender	Male	7 (46.67%)	40 (57.14%)
	Female	8 (53.33%)	30 (42.86%)
WBC count	Normal	15 (100%)	44 (62.86%)
	Increased	0 (0%)	23 (32.86%)
	Decreased	0 (0%)	3 (4.29%)
NU count	Normal	15 (100%)	69 (98.57%)
	Increased	0 (0%)	1 (1.43%)
LY count	Normal	15 (100%)	41 (58.57%)
	Decreased	0 (0%)	29 (41.43%)
EO count	Normal	14 (93.33%)	26 (37.14%)
	Increased	0 (0%)	3 (4.29%)
	Decreased	1 (6.67%)	41 (58.57%)
Plat. count	Normal	15 (100%)	60 (85.71%)
	Increased	0 (0%)	6 (8.57%)
	Decreased	0 (0%)	4 (5.71%)
HB level	Normal	14 (93.33%)	56 (80%)
	Decreased	1 (6.67%)	14 (20%)
AST level	Normal	15 (100%)	48 (68.57%)
	Increased	0 (0%)	22 (31.43%)
ALT level	Normal	15 (100%)	49 (70%)
	Increased	0 (0%)	21 (30%)
S. Cr level	Normal	15 (100%)	59 (84.29%)
	Increased	0 (0%)	10 (14.29%)
	Decreased	0 (0%)	1 (1.43%)
BUN level	Normal	15 (100%)	39 (55.71%)
	Increased	0 (0%)	30 (42.86%)
	Decreased	0 (0%)	1 (1.43%)

**Table 4:** Comparison of the percentage of total CD3^+^ T cells and different T cell subtypes, Th CD4^+^, Tc CD8^+^, and Treg CD4^+^ CD25^+^, between different COVID-19 patient groups and controls.

T Cell Subtypes	Controls	Mild/Asymptomatic Cases	Moderate Cases	Severe/Critical Cases
CD3 Cells % Mean ± SD	41.15 ± 4.823	23.45 ± 2.701	22.49 ± 2.894	17.55 ± 2.083
CD4 Cells % Mean ± SD	39.11 ± 4.488	20.11 ± 2.026	20.11 ± 2.026	16.08 ± 1.734
CD8 Cells % of Mean ± SD	20.85 ± 3.201	12.22 ± 2.302	8.868 ± 1.304	5.492 ± 0.7858
CD4CD25 Cells % Mean ± SD	21.94 ± 3.517	9.500 ± 1.205	7.718 ± 1.022	6.592 ± 0.8424
CD38 Cells % of Mean ± SD	55.38 ± 6.092	44.10 ± 3.331	43.83 ± 4.186	39.13 ± 2.821
CD4CD38 Cells % Mean ± SD	36.93 ± 3.868	24.42 ± 2.136	19.46 ± 2.330	16.61 ± 1.782
CD8CD38 Cells % Mean ± SD	24.85 ± 3.501	11.31 ± 1.278	13.32 ± 1.313	11.85 ± 0.9256

**Table 5:** Comparison of age, laboratory data, and CD38 expression in CD4^+^ T cells and CD8^+^ T cells between different patient groups.

	Mild/Asymptomatic Cases	Moderate Cases	Severe/Critical Cases		
	Mean ± SD	Mean ± SD	Mean ± SD	F	*p*-Value
Age	38.23 ± 8.09	52.68 ± 14.86	60.5 ± 14.07	18.324	<0.001 ^a^
WBCs K/UL	7.56 ± 2.49	7.07 ± 3.77	12.66 ± 5.74	12.565	<0.001 ^b^
NU K/UL	4.52 ± 2	5.63 ± 3.54	11.47 ± 5.37	21.371	<0.001 ^b^
LY K/UL	2.49 ± 0.64	1.16 ± 0.63	0.91 ± 0.95	27.933	<0.001 ^a^
NU/LY	1.81 ± 0.58	5.71 ± 4.21	20.06 ± 18.26	17.33	<0.001 ^b^
EO K/UL	0.27 ± 0.24	0.06 ± 0.14	0.1 ± 0.35	4.046	0.022 ^c^
Plat. K/UL	290.68 ± 82.98	285.05 ± 86.21	311.73 ± 133.88	0.432	0.651
HB gm/dL	12.95 ± 1.55	12.24 ± 1.81	12.65 ± 2.21	0.784	0.461
AST U/L	25.64 ± 9.82	47.38 ± 34.11	70.62 ± 47.51	9.775	<0.001 ^d^
ALT U/L	23.23 ± 8.53	55.18 ± 60.51	62.58 ± 40.72	5.634	0.005 ^a^
S.Cr Umol/L	62.59 ± 14.79	73.24 ± 37.37	106.77 ± 51.32	8.668	<0.001 ^b^
BUN mmol/L	3.47 ± 1.53	5.39 ± 2.31	10.91 ± 6.28	21.235	<0.001 ^b^
CD38 exp. on CD4^+^ T cells (MFI) × 10^3^	88.00 ± 13,947.66	96.94 ± 19,301.2	125.64 ± 24,802.79	23.147	<0.001 ^b^
CD38 exp. on CD8^+^ T cells (MFI) × 10^3^	67.74 ± 14,828.01	78.52 ± 23,801.7	125.77 ± 24,581.98	49.259	<0.001 ^b^

*p*-value < 0.05 is considered significant, *p*-value < 0.01 is considered very significant, *p*-value < 0.001 and *p*-value < 0.0001 are considered extremely significant. Post hoc Bonferroni test: ^a^ Mild vs. Moderate (S), Mild vs. Severe (S), and Moderate vs. Severe (NS); ^b^ Mild vs. Moderate (NS), Mild vs. Severe (S), and Moderate vs. Severe (S); ^c^ Mild vs. Moderate (S), Mild vs. Severe (NS), and Moderate vs. Severe (NS); ^d^ Mild vs. Moderate (NS), Mild vs. Severe (S), and Moderate vs. Severe (NS).

**Table 6:** Comparison of the gender, laboratory data distribution, and mortality between different patient groups.

		Mild/Asymptomatic Cases	Moderate Cases	Severe/Critical Cases		
		N (%)	N (%)	N (%)	Value	*p*-Value
Gender	Male	5 (22.73%) ^a^	13 (59.09%) ^b^	22 (84.62%) ^b^	X2 = 18.69	<0.001
	Female	17(77.27%) ^a^	9 (40.91%) ^b^	4 (15.38%) ^b^		
WBC count	Normal	18 (81.82%) ^a^	17 (77.27%) ^a^	9 (34.62%) ^b^	Fisher exact test	0.001
	Increased	3 (13.64%) ^a^	4 (18.18%) ^a^	16 (61.54%) ^b^		
	Decreased	1 (4.55%) ^a^	1 (4.55%) ^a^	1 (3.85%) ^a^		
NU count	Normal	22 (100%)	22 (100%)	25 (96.15%)	Fisher exact test	1.000
	Increased	0 (0%)	0 (0%)	1 (3.85%)		
LY count	Normal	22 (100%) ^a^	15 (68.18%) ^b^	4 (15.38%) ^c^	X2 = 36.38	<0.001
	Decreased	0 (0%) ^a^	7 (31.82%) ^b^	22 (84.62%) ^c^		
EO count	Normal	18 (81.82%) ^a^	6 (27.27%) ^b^	2 (7.69%) ^b^	Fisher exact test	<0.001
	Increased	2 (9.09%) ^a^	0 (0%) ^a^	1 (3.85%) ^a^		
	Decreased	2 (9.09%) ^a^	16 (72.73%) ^b^	23 (88.46%) ^b^		
Plat. count	Normal	21 (95.45%) ^a^	21 (95.45%) ^a^	18 (69.23%) ^b^	Fisher exact test	0.027
	Increased	0 (0%) ^a^	1 (4.55%) ^a^	5 (19.23%) ^b^		
	Decreased	1 (4.55%) ^a^	0 (0%) ^a^	3 (11.54%) ^b^		
HB level	Normal	21 (95.45%)	16 (72.73%)	19 (73.08%)	Fisher exact test	0.072
	Decreased	1 (4.55%)	6 (27.27%)	7 (26.92%)		
AST level	Normal	22 (100%) ^a^	15 (68.18%) ^b^	11 (42.31%) ^b^	X2 = 18.41	<0.001
	Increased	0 (0%) ^a^	7 (31.82%) ^b^	15 (57.69%) ^b^		
ALT level	Normal	22 (100%) ^a^	16 (72.73%) ^b^	11 (42.31%) ^b^	X2 = 19	<0.001
	Increased	0 (0%) ^a^	6 (27.27%) ^b^	15 (57.69%) ^b^		
S. Cr level	Normal	22 (100%) ^a^	19 (86.36%) ^a,b^	18 (69.23%) ^b^	Fisher exact test	0.005
	Increased	0 (0%) ^a^	2 (9.09%) ^a,b^	8 (30.77%) ^b^		
	Decreased	0 (0%) ^a^	1 (4.55%) ^a^	0 (0%) ^a^		
BUN level	Normal	20 (90.91%) ^a^	15 (68.18%) ^a^	4 (15.38%) ^b^	Fisher exact test	<0.001
	Increased	2 (9.09%) ^a^	6 (27.27%) ^a^	22 (84.62%) ^b^		
	Decreased	0 (0%) ^a^	1 (4.55%) ^a^	0 (0%) ^a^		
Mortality	Survivors	22 (100%) ^a^	21 (95.45%) ^a^	15 (57.69%) ^b^	Fisher exact test	<0.001
	Deceased	0 (0%) ^a^	1 (4.55%) ^a^	11 (42.31%) ^b^		

Post Hoc Bonferroni Test: column proportions with the same superscript (lowercase letters) do not differ significantly from each other at the 0.05 level.

**Table 7:** Correlation between CD38 expression in Th CD4^+^ T cell and Tc CD8^+^ T cell subtypes.

		CD38/CD8^+^ Cells
CD38/CD4^+^ cells	r	0.583
	*p*-value	<0.001
	Sig	S

**Table 8:** ROC analysis of CD38/CD4^+^ cells and CD38/CD8^+^ cells to predict mortality among patients of the three studied groups.

Variable	AUC	SE	95% CI
CD38 expression in CD4^+^ T cells	0.801	0.06	0.687 to 0.887
CD38 expression in CD8^+^ T cells	0.834	0.05	0.725 to 0.913

**Table 9:** The cut-off value for CD38 expression (MFI × 10^3^) in Th CD4^+^ T cell and Tc CD8^+^ T cell subtypes as a predictor of mortality among patients of the three studied groups.

	Predictive Value				
	Cutoff	Sensitivity %	Specificity %	+PV	−PV
CD38 expression in CD4^+^ T cells MFI (× 10^3^)	>100.87	100	56.14	34.2	100
CD38 expression in CD8^+^ T cells MFI (× 10^3^)	>91.46	100	67.86	41.9	100

**Table 10:** ROC analysis of CD38/CD4^+^ cells and CD38/CD8^+^ cells to predict mortality among patients of the moderate group.

Area Under the Curve					
Test Result Variable(s)	Area	Std. Error	Asymptotic Sig. *	Asymptotic 95% Confidence Interval	
				Lower Bound	Upper Bound
CD38/CD4^+^ cells MFI	0.619	0.106	0.694	0.411	0.827
CD38/CD8^+^ cells MFI	0.714	0.099	0.478	0.521	0.908

Under the nonparametric assumption; * Null hypothesis: true area = 0.5.

**Table 11:** ROC analysis of CD38/CD4^+^ cells and CD38/CD8^+^ cells to predict mortality among patients of the severe/critical group.

Area Under the Curve					
Test Result Variable(s)	Area	Std. Error	Asymptotic Sig. *	Asymptotic 95% Confidence Interval	
				Lower Bound	Upper Bound
CD38/CD4^+^ cells MFI	0.548	0.118	0.681	0.316	0.779
CD38/CD8^+^ cells MFI	0.440	0.118	0.607	0.210	0.671

Under the nonparametric assumption; * Null hypothesis: true area = 0.5.

## Data Availability

The data are not publicly available for patients’ privacy.
